# Enhancement of Signal-to-Noise Ratio of Void Detection Signals in Concrete-Filled Steel Tubular Structures Using the Good Point Set and Vibrational Snow Ablation Optimizer

**DOI:** 10.3390/s26134261

**Published:** 2026-07-04

**Authors:** Gen He, Zhongchu Tian, Fanbo Guo, Jiaqi Chen, Binlin Xu

**Affiliations:** 1 College of Civil and Construction Engineering, Hunan Institute of Technology, Hengyang 421002, China; 2 School of Civil Engineering, Fujian University of Technology, Fuzhou 350118, China

**Keywords:** concrete-filled steel tube (CFST), void detection, good point set and vibrational snow ablation optimizer (GVSAO), gram angle field (GAF), signal-to-noise ratio (SNR)

## Abstract

Deep learning (DL)-based percussion methods in concrete-filled steel-tube (CFST) void detection have gained much attention. However, the detection signal contains a large amount of noise, which affects the accuracy of qualitative and quantitative analyses of the subsequent detection results. To improve the signal-to-noise ratio (SNR) during percussion detection, this study proposes a CFST void detection method using the good point set and vibrational snow ablation optimizer (GVSAO) algorithm and dual-channel parallel convolutional neural networks (CNNs). The proposed method employs the gram angle field (GAF) to transform percussive sound signals into images. It then constructs a dual-channel parallel CNN structure, where the GAF is decomposed into the following two maps: the gram angle sum field (GASF) and the gram angle difference field (GADF). These maps are simultaneously fed into the CNN for training. The outputs from the two channels are concatenated and fused. Finally, the GVSAO algorithm was used for model optimization to improve convergence speed and recognition accuracy. Both the temporal and spatial characteristics of the knocking sound signal are fully preserved, while the interference of different construction noises is effectively avoided. Validation experiments were conducted on CFST specimens with different heights of voids (0, 50, 100, and 150 mm) under different pressure loads. The original sample dataset and the signal-enhanced dataset were obtained by adding background noise with different SNRs. The test results show that the prediction accuracies on the original signal dataset are consistently above 98.74%. Among them, the accuracy achieves 100% at pressure loads of 0 and 50 tons. Additionally, the prediction accuracies on the signal-enhanced dataset are all above 97.2%, indicating that the model maintains a high level of classification performance. This suggests that the model can effectively suppress noise and exhibits excellent robustness.

## 1. Introduction

To comprehensively leverage the advantages of steel and concrete materials, engineers have incorporated these two materials into the same cross-sections of bridge structures. This resulted in the creation of concrete-filled steel-tubular (CFST) arch bridges [1,2]. CFST arch bridges capitalize on the characteristics of composite materials and arch structures [3]. Globally, approximately 500 CFST arch bridges have been constructed [4]. Nevertheless, in the context of steel–concrete composite structures, which entail the integration of internal and external structural elements, the performance of the steel–concrete interface is solely dependent on the adhesion between the steel tube and the concrete. Owing to the lack of mechanical interlocking or other forms of direct connection between these two materials, engineers have expressed concerns about potential problems such as debonding and void formation, which may ultimately undermine the integrity and functionality of the steel–concrete assembly [5,6]. Consequently, it is imperative to develop effective void detection methods to guarantee the safety and integrity of the CFST structures.

Currently, the methods for detecting voids in concrete-filled steel tube (CFST) structures can be classified into non-destructive testing (NDT) and destructive testing (DT). Destructive testing involves core drilling and sampling to directly expose the state of the voids, which may cause certain damage to the structure [7]. Non-destructive testing (NDT) techniques are now regarded as a promising alternative for void detection [8]. Presently, the commonly employed NDT techniques encompass percussion detection, ultrasonic detection, scanning technique detection, X-ray detection, elastic wave detection, infrared thermography detection, and radar detection [9,10,11,12,13,14,15,16]. Among these, the percussion testing method is one of the most convenient, rapid, and easily implementable approaches. Nevertheless, this method is highly reliant on the operator’s expertise, and the results may display a certain degree of subjectivity, thus being prone to misjudgment and omission. Consequently, it severely restricts the development of the application of the percussion detection method [17,18].

In recent years, instruments and equipment, such as percussion hammers, sensors, microphones, and collectors, have been introduced and utilized in percussion inspections. These have enhanced the inspection accuracy by converting the sound signals generated during percussion into electrical signals. The modern theory of localized percussion detection was put forward by Cawlwy and Adams. Time-domain and frequency-domain analyses are conducted on the stress signals obtained from percussion. As the defect size increases, the impact duration lengthens, the frequency decreases, and the stress amplitude increases [19,20]. Subsequently, WichiTech, Rolls-Royce Mateval, and Mitsui developed products for detecting defects in composite materials, such as RD3, woodpecker, and Tapometer. P. K. Raju proposed the AIT method and applied the acoustic emission probe in a percussion device [21,22]. Wu et al. [23,24] conducted computational analysis and processing on acceleration, acoustic, and pressure signals generated during the percussion process. In recent years, deep learning (DL) has experienced significant development, providing novel perspectives and methodologies for sound signal recognition.

Deep learning (DL) diagnostic methods obviate the need for precise baseline signals or accurate modeling. By mining a substantial volume of data within a specific class of features as the basis for fault judgment, these methods exhibit strong robustness and portability. They have been extensively applied in the field of defect diagnosis [25,26,27,28,29]. Jiang et al. [30] developed a structure where multiple percussion devices are simultaneously actuated to enhance detection efficiency. To expand the detection scope, a regionally integrated bispectral method is proposed for extracting feature vectors. Based on the disparity in the bispectral frequency distributions of dense and void signals, a thresholding approach is proposed for classifying the two scenarios. The classification accuracy surpasses that of the traditional power spectrum method; however, the detection accuracy remains relatively low.

Kang et al. [31] estimated the size of the void using the following four dimensions: the measured area energy under the rectified signal envelope of the acquired acoustic signal (MARSE), the slope of the initial portion of the frequency-domain migration spectrum, the peak amplitude of the wavelet transform, and the frequency corresponding to the peak amplitude. The accuracy of both the peak amplitude of the wavelet transform for nulling and the frequency corresponding to this peak is maintained at a high level. Nevertheless, the influence of background noise has not been considered.

Chen et al. [32] analyzed the sound wave data of concrete wall thickness voids of different sizes in steel tubes through power spectral density analysis. They extracted 9 features for training in the decision tree (DT) and support vector machine (SVM), with detection accuracies of 96.33% and 94.17% respectively. To enhance the detection accuracy, they employed the class activation mapping (CAM) to classify the CFST structural void percussion sound signal features. The defect detection accuracy reached 99.81%, yet there was a decline in computational efficiency [33]. To account for the effect of background noise, they proposed a lightweight CFST void detection method utilizing the Mel Frequency Cepstrum Coefficient (MFCC) algorithm and integrated machine learning (ML). This method improves the detection accuracy and efficiency, as well as the robustness, of signal processing [34].

In this study, a void detection method for CFST structures based on the combination of the GVSAO algorithm and dual-channel parallel CNN is proposed. The method is inspired by GAF dense feature extraction [35] and deep learning [36]. The CFST specimens were first percussed by tapping them in the laboratory to obtain their void sound signals under different pressure loads. The signals are divided into the following four types: “no voids” and voids with three different height levels (5 cm, 10 cm, and 15 cm). Enhanced data were generated by introducing background noise to the original signals and subsequently labeled for further processing. The labeled one-dimensional (1D) temporal signals were transformed into two-dimensional (2D) images of two types, GASF and GADF, using the GAF method. These images were then fed into two parallel CNNs for feature extraction and fusion. Finally, classification optimization is performed based on the GVSAO algorithm to obtain an accurate void diagnosis model. The performance of the proposed void diagnostic model is first validated using experimental data. Subsequently, the diagnostic accuracy and efficiency of different SNR models were analyzed by extracting signal features. Overall, the proposed method has the following two practical properties:

(i) *The proposed method ensures the complete preservation of both temporal and spatial characteristics of the percussion sound signal while effectively mitigating interference from various construction noises.*
(ii) *The proposed method has the effect of visualizing and processing signal features in a certain way.*

The rest of the paper is organized as follows: Section 2 describes the CFST structural void detection method, including GAF conversion, training of dual-channel parallel CNNs, GVSAO, and its extension in this study. Section 3 details the experimental steps. Section 4 discusses the prediction results. The discussion is presented in Section 5. The conclusions are presented in Section 6.

## 2. CFST Structural Void Detection Method

**Figure 1:**
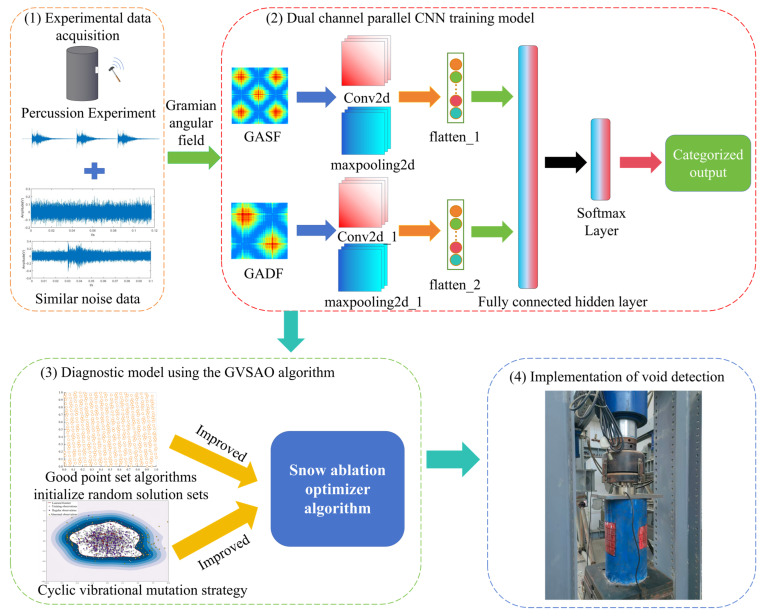
Conceptual flowchart of the CFST structure void detection methodology.

To enhance the SNR during percussion detection, Figure 1 shows the conceptual flowchart of the proposed GVSAO algorithm in this study, which is integrated with parallel CNNs for void detection in the CFST structure. The procedure is divided into the following four steps: (1) experimental data acquisition; (2) construction of a dual-channel parallel CNN training model; (3) development of a diagnostic model based on the GVSAO algorithm; and (4) implementation of void detection.

The procedure for detecting cavities in CFST structures is outlined as follows: (1) A hammer is utilized to percuss the surface of the steel tube, and the resulting audio signal is recorded using a smartphone. The recorded signals are subsequently classified and stored on the device. Various background noises are introduced into the percussive signals to elevate the noise level, thereby enhancing the robustness of the model against environmental interference. (2) The GASF graph and GADF graph are simultaneously fed into two parallel CNNs. After undergoing double-layer convolution and pooling operations, each CNN outputs a 1D vector. These two sets of 1D vectors are then concatenated and fused. The fused features are subsequently passed through a fully connected layer into a Softmax classifier, which outputs the probabilities of detecting different defective states of the CFST structure based on the four percussive signals. (3) To establish an accurate diagnostic model, the GVSAO algorithm is employed to optimize the hyperparameters. This optimization process improves the diagnostic accuracy for identifying various defect states in CFST structures. (4) Laboratory experiments are conducted to detect the defective states of the CFST structures.

### 2.1. Enhanced Signal Robustness

**Figure 2:**
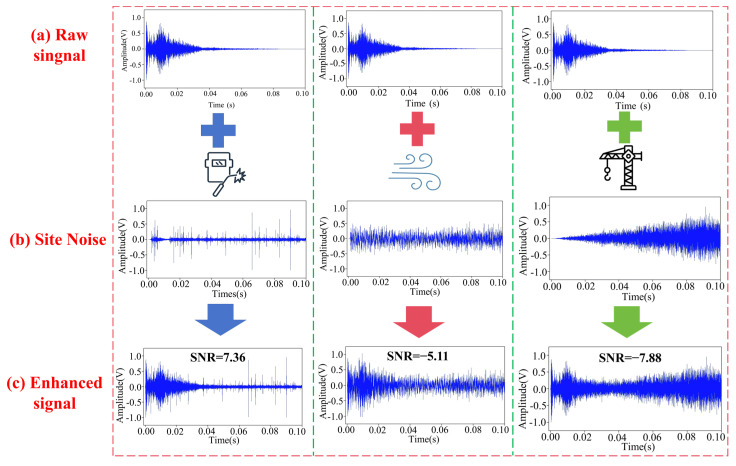
Construction site noise added: (**a**) original signal; (**b**) construction site noise; (**c**) enhanced signal.

In engineering applications, the acquisition of sound signals is often subject to a certain degree of noise interference. High diagnostic accuracy, especially for sound signals, in the face of the interference of various sounds in the natural environment. It is what will show the superiority of the testing method. Therefore, the test set samples were augmented with the mechanical equipment, wind, and welding noise disturbances in the construction site, as shown in Figure 2 (during recording, only isolated noises from mechanical equipment, wind, and welding were present, with no other noise interference). Test samples are fed into each trained model to verify the good robustness of the method proposed in this paper. The following equation from the paper published by Shen [34] et al. was used to describe the addition of noise:

(1) $$A_{E}^{i}=A_{raw}^{i}+A_{noise}^{i}\left(i=1,2,\ldots ,n\right),$$

where *i* is the signal point and *n* is the number of signal points. *A_E_*, *A_raw_*, and *A_noise_* denote the amplitude of the enhanced sound signal, the amplitude of the original sound signal, and the amplitude of the noisy sound signal, respectively. The degree of noise level after the enhancement of the sound signal can be quantitatively represented and calculated using the SNR, as shown in the formula below [34]:

(2) $$\mathrm{SNR}\left(\mathrm{dB}\right)=10\;\log_{10}\left(\frac{\sum_{i=1}^{N}{\left(A_{raw}^{i}\right)}^{2}/N}{\sum_{i=1}^{N}{\left(A_{noise}^{i}\right)}^{2}/N}\right),$$

The values of the SNR under different background noises are obtained from the above equation as 7.36 dB, −5.11 dB, and −7.88 dB, as shown in Figure 2c. In addition, to demonstrate the efficacy of the proposed method in enhancing the SNR, Gaussian white noise with SNR levels of −10 dB, −5 dB, 0 dB, 5 dB, and 10 dB was selected for evaluation. These samples were used as inputs for the test set and subsequently fed into the optimized diagnostic model.

### 2.2. Signal Feature Extraction

#### 2.2.1. GAF−Based Data Visualization

GAF converts 1D sound signals into 2D images. Its fundamental principle is to transform 1D data from a Cartesian coordinate system to a polar coordinate system, based on its unique form of inner product definition. The time−dependent information is characterized through trigonometric summation and difference operations. Sequential tiling into the image from top left to bottom right generates the following two types of images: GASF and GADF.

Defining a time−series $Z=\left\{z_{1},z_{2},\cdots ,z_{i},\cdots ,z_{n}\right\}$, where *i* is the time point, *i* = 1, 2, ..., *n*. The process of transforming ***Z*** to GAF is as follows [35]:

(1) Standardized scaling: the time−series ***Z*** in the Cartesian coordinate system is first scaled to ${\tilde{z}}_{i}$ in the range of [−1, 1] using Equation (1).
(2) Polar coordinate transformations: The polar coordinate transformation converts the time-series magnitude data into vectors and subsequently performs the inner product operation. Its transformation formula is as follows:

(3) $$\left\{\begin{array}{l} \begin{array}{cc} \phi =\arccos{\tilde{z}}_{i} & -1\leq {\tilde{z}}_{i}\leq 1,{\tilde{z}}_{i}\in \tilde{Z} \end{array}\\ \begin{array}{cc} r=t_{i}/N & t_{i}=1,2,\cdots ,N \end{array} \end{array}\right.,$$

where *t_i_* is the time point; *N* is a constant factor of the regularized polar coordinate generating space; *ϕ* is the phase angle; *r* is the polar coordinate radius; $\tilde{Z}$ is the normalized scaled ***Z***; $\tilde{Z}$ is transformed in polar coordinates to form a sequence of polar coordinate vectors, $\dot{Z}=\left\{{\dot{z}}_{1},{\dot{z}}_{2},\cdots ,{\dot{z}}_{i},\cdots ,{\dot{z}}_{n}\right\}$.

The above mapping equation has two important properties: First, it enables bi-directional mapping. According to the trigonometric properties, cos*ϕ* is monotonically decreasing at $\phi \in \left[0,\pi \right]$. Thus, given the instantaneous value of the temporal amplitude, the corresponding value mapped to polar coordinates is uniquely determined, and the inverse mapping is also unique. Second, absolute time relations are preserved in polar coordinates.

#### 2.2.2. GAF Transform

Define the Gram matrix, ***G***, as shown in Equation (4). The inner product between different vectors can characterize their correlation, while the angle between these vectors shows the degree of correlation among them.

(4) $$G=Z^{T}Z=\left[\begin{array}{ccc} \langle {\dot{z}}_{1},{\dot{z}}_{1}\rangle & \cdots & \langle {\dot{z}}_{1},{\dot{z}}_{n}\rangle \\ \langle {\dot{z}}_{2},{\dot{z}}_{1}\rangle & \ddots & \langle {\dot{z}}_{2},{\dot{z}}_{n}\rangle \\ \vdots & & \vdots \\ \langle {\dot{z}}_{n},{\dot{z}}_{1}\rangle & \cdots & \langle {\dot{z}}_{n},{\dot{z}}_{n}\rangle \end{array}\right],$$

where $\langle \cdot ,\cdot \rangle$ denotes the inner product operation.

GAF defines two unique forms of inner product with penalty terms to eliminate the effect of Gaussian noise, which can be described as follows:

(5) $$\langle {\dot{z}}_{i},{\dot{z}}_{j}\rangle =\cos\left(\phi_{i}+\phi_{j}\right),$$

(6) $$\langle {\dot{z}}_{i},{\dot{z}}_{j}\rangle =\sin\left(\phi_{i}+\phi_{j}\right),$$

where $\phi_{i}$ and $\phi_{j}$ are the phase angles of ${\dot{z}}_{i}$ and ${\dot{z}}_{j}$, respectively.

For the above two definitions of inner product forms, two corresponding GAFs can be obtained, namely, GASF and GADF, respectively. Their Gram matrices are ***G***_GASF_ and ***G***_GADF_, respectively:

(7) $$G_{GASF}=\left[\begin{array}{ccc} \cos\left(\phi_{1}+\phi_{1}\right) & \cdots & \cos\left(\phi_{1}+\phi_{n}\right)\\ \cos\left(\phi_{2}+\phi_{1}\right) & \ddots & \cos\left(\phi_{2}+\phi_{n}\right)\\ \vdots & & \vdots \\ \cos\left(\phi_{n}+\phi_{1}\right) & \cdots & \cos\left(\phi_{n}+\phi_{n}\right) \end{array}\right],$$

(8) $$G_{GADF}=\left[\begin{array}{ccc} \sin\left(\phi_{1}+\phi_{1}\right) & \cdots & \sin\left(\phi_{1}+\phi_{n}\right)\\ \sin\left(\phi_{2}+\phi_{1}\right) & \ddots & \sin\left(\phi_{2}+\phi_{n}\right)\\ \vdots & & \vdots \\ \sin\left(\phi_{n}+\phi_{1}\right) & \cdots & \sin\left(\phi_{n}+\phi_{n}\right) \end{array}\right],$$

**Figure 3:**
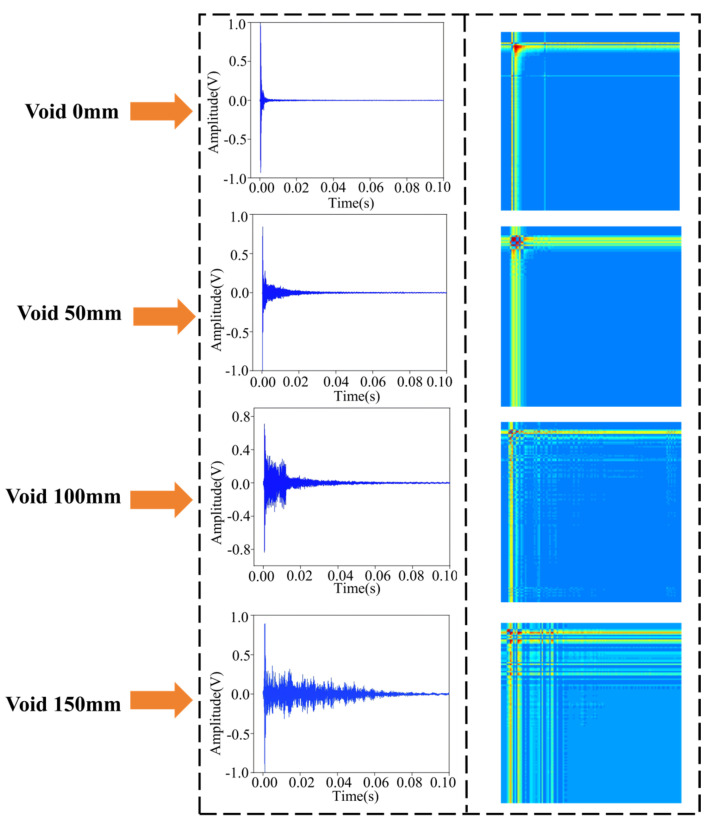
Characterization of the GAF transformation for different heights of the void state.

After transforming the Gram angle field, as described above, the characterization results of the CFST structure at different heights of the void state are obtained, as shown in Figure 3.

For sound amplitude data that are close together on the time scale, the GAF transform effectively mitigates signal interference. Let Δ*ϕ_i_* and Δ*ϕ_j_* represent the phase-angle offsets of vectors ${\dot{z}}_{i}$ and ${\dot{z}}_{j}$, respectively, under noise interference. In the presence of differential mode noise interference, the phase-angle offset satisfies Δ*ϕ_i_* = −Δ*ϕ_j_*. When there is a common mode noise disturbance, the relationship Δ*ϕ_i_* = Δ*ϕ_j_* holds. These two different types of interference can be automatically eliminated by substituting the corresponding interference terms into Equation (9) and Equation (10), respectively.

(9) $$\langle {\dot{z}}_{i},{\dot{z}}_{j}\rangle =\cos\left[\left(\phi_{i}+\Delta \phi_{i}\right)+\left(\phi_{j}+\Delta \phi_{j}\right)\right],$$

(10) $$\langle {\dot{z}}_{i},{\dot{z}}_{j}\rangle =\sin\left[\left(\phi_{i}+\Delta \phi_{i}\right)-\left(\phi_{j}+\Delta \phi_{j}\right)\right],$$

### 2.3. Training Process Using Dual-Channel Parallel CNNs

This paper adopts a dual-channel parallel CNN for training. First, the GASF and GADF maps are simultaneously input into two parallel CNN channels. After double-layer convolution-pooling, each CNN channel outputs a set of 1D vectors. Then, the two sets of 1D vectors are concatenated and fused to form a comprehensive feature representation. The fused features are finally fed into the Softmax classifier through the fully connected layer, which predicts the probabilities of the four CFST structures being in various defective states. The dual-channel parallel CNN structure is shown in Figure 1, and the network structure parameters are provided in Table 1.

### 2.4. Diagnostic Model Construction Based on the GVSAO Algorithm

**Figure 4:**
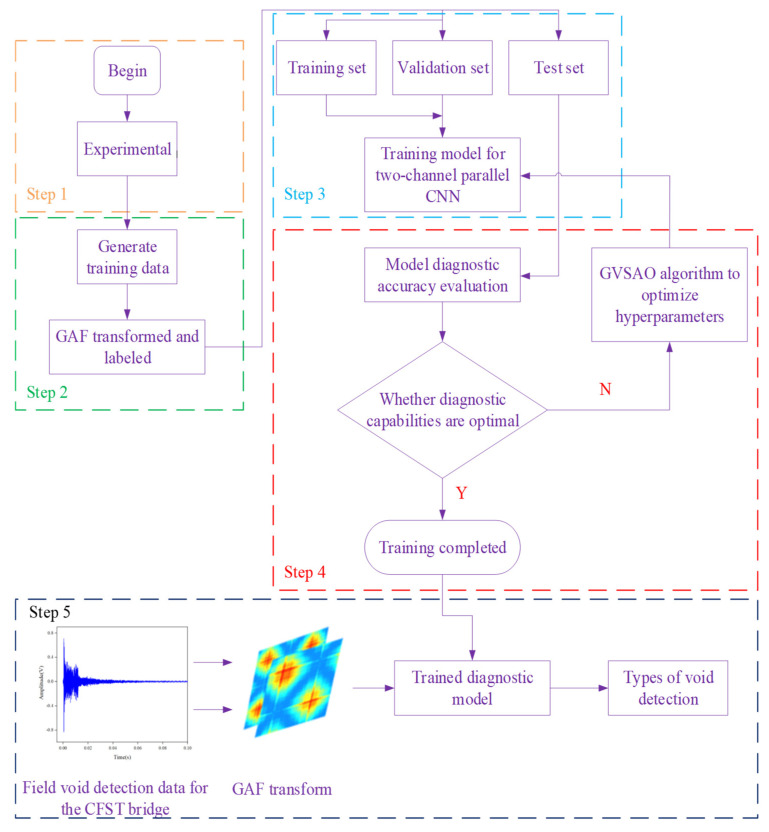
CFST void detection overall flowchart.

To improve the accuracy of the CFST defect diagnosis, this section introduces the application of the GVSAO algorithm for optimizing hyperparameters to identify the optimal diagnostic model. The overall flowchart is shown in Figure 4.

The process of optimizing the diagnostic model by the GVSAO algorithm is described in detail as follows:

The snow ablation optimizer (SAO) algorithm draws its inspiration from the sublimation and melting behavior of snow, encompassing four primary phases: the initialization of the sample data, the exploration phase, the exploitation phase, and the two-population mechanism. A key characteristic of this algorithm is its ability to balance exploitation and exploration within the solution space, thereby preventing premature convergence of diagnostic models. To leverage the strengths of this algorithm for the extraction of abnormal sound signal features and the search for optimal diagnostic models, this section proposes an optimization and enhancement strategy by incorporating good point sets and vibrational strategies as follows:

(1) *Good point set algorithms initialize random solution sets*

The good point set algorithm was first proposed by Hua [37]. Suppose there exists such a set in Euclidean space *H_s_* of dimension *s*. $P_{n}\left(k\right)=\left\{\left(\left\{r_{1}^{\left(n\right)}\cdot k\right\},\left\{r_{2}^{\left(n\right)}\cdot k\right\},\cdots ,\left\{r_{s}^{\left(n\right)}\cdot k\right\}\right),1\leq k\leq n\right\}$. The deviation meets $\varphi \left(n\right)=C\left(r,\varepsilon \right)n^{-1+\varepsilon }$. We refer to this set as the set of good points. *r* is a good point. The good point is $r^{i}=\left\{2\cos\left(\frac{2\pi i}{p}\right)\right\};1\leq i\leq n$, where *p* is the smallest prime number satisfying p≥2∗s+3. The set of good points can be mapped to the search space by the following equation [38]:

(11) $$Z=\left(U-L\right)\left\{P_{n}\left(k\right)\right\}+L={\left[\begin{array}{ccccc} z_{1,1} & z_{1,2} & \cdots & z_{1,Dim-1} & z_{1,Dim}\\ z_{2,1} & z_{2,2} & \cdots & z_{2,Dim-1} & z_{2,Dim-1}\\ \vdots & \vdots & \vdots & \vdots & \vdots \\ z_{N-1,1} & z_{N-1,2} & \cdots & z_{N-1,Dim-1} & z_{N-1,Dim}\\ z_{N,1} & z_{N,2} & \cdots & z_{N,Dim-1} & z_{N,Dim} \end{array}\right]}_{N\times Dim},$$

where *U* and *L* are the upper and lower bounds of the solution space, respectively. *N* is the swarm size. *Dim* is the dimension of the solution space and also corresponds to the number of hyperparameters.

**Figure 5:**
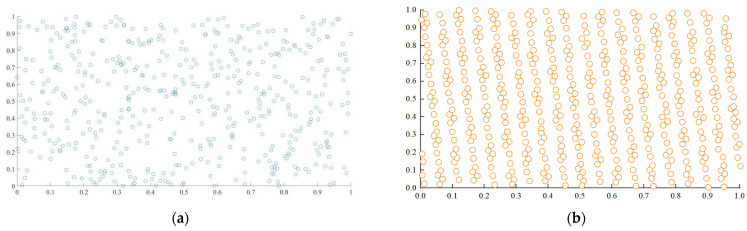
The set of points in two-dimensional space: (**a**) random strategy generates the point set; (**b**) good point set.

To reflect the state of the art of the good point set algorithm, this section generates 500 sample points using both the good-point-set strategy and the randomized strategy. Figure 5a shows the initial overall distribution of 500 points generated by the good point set in two-dimensional space. Figure 5b shows the initial overall distribution of 500 points generated by the random strategy.

As can be seen from Figure 5, the points generated by the good point set are more uniform than those generated through random methods. Therefore, the population diversity can be effectively enhanced by introducing the good point set, which helps the algorithm to get rid of local optimality.

(2) *Periodic oscillatory mutation policy search for optimal solutions*

Implementing a generalized mutation operation on the random solution set after initializing the good-point-set strategy can be described as follows [39]:

(12) $$z_{i,j}\left(t\right)=F\left(g\left(z_{i,j}\left(t\right)\right),fr\right),$$

where *F* is the generalized mutation function. It is the number of iterations. $g\left(z_{i,j}\left(t\right)\right)$ is the mutation operator that provides the progeny carrier. *fr* is a customized oscillatory fluctuation frequency. Mutation strategies focus on how to apply mutation operators in the optimization process. After updating the program and then applying the mutation operator to all ice crystal dimensions of the entire swarm in each *fr*^−1^ period, the particles in the swarm diffuse throughout the design space. The value in this article is 10. This operator is called the global mutation operator and is given by the following [40]:

(13) $$\begin{array}{l} z_{i,j}\left(t\right)=z_{i,j}\left(t\right)\left[1+A\left(0.5-rand\right)\delta \right]\\ i=1,2,\ldots ,N\\ j=1,2,\ldots ,dim\\ \delta =\left\{\begin{array}{l} 1\;\;\;if\;\;\;t=nfr\;\;\;n=1,2,\ldots \\ 0\;\;\;if\;\;\;t\neq nfr \end{array}\right. \end{array},$$

where *A* is the customized oscillatory fluctuation amplitude, which can be a fixed value or a dynamic value that changes with iterations. The value in this article is 5. The *rand* is a random number generated by a random number generator in the interval [0, 1].

(3) *Dual population mechanism balances computational accuracy and efficiency*

**Figure 6:**
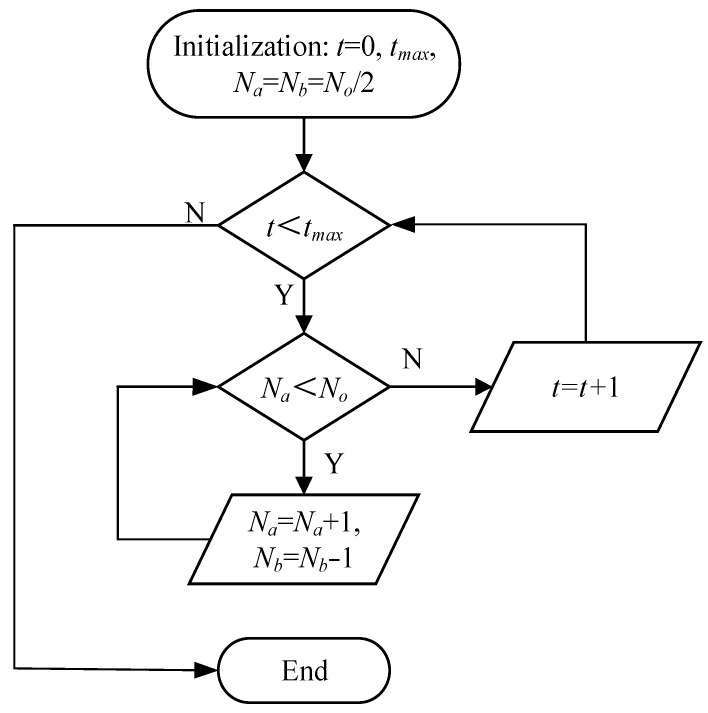
Iterative flowchart for the two-population mechanism.

The GVSAO algorithm was designed with a two-population mechanism to address the need for both exploration and exploitation, and it remains an area of ongoing research and refinement. In the initial stages of the iteration process, the entire population is randomly divided into two equal-sized subpopulations. The whole population and these two subpopulations are denoted as *P_o_*, *P_a_*, and *P_b_*, respectively. Furthermore, the sizes of *P_o_*, *P_a_*, and *P_b_* are denoted as *N_o_*, *N_a_*, and *N_b_*, respectively. Herein, *P_a_* is responsible for exploration, while *P_b_* focuses on exploitation. During the iteration process, *P_b_* decreases and *P_a_* increases. The detailed iterative procedure is shown in Figure 6.

To develop multiple search paths to address the highly dispersed extension of the sample data, this paper introduces one of the most classical snowmelt models, the degree-day method [41], to reflect this situation. The general form of this approach is as follows:

(14) M=DDF×T,

where *M* is the snowmelt rate, which is the key parameter for modeling the snowmelt behavior of the development path. *T* is the average daily temperature. *DDF* denotes the degree-day factor ranging from 0.35 to 0.6 [40]. The updated *DDF* value during each iteration can be expressed as follows:

(15) $$DDF=0.35+0.25\times \frac{e^{\frac{t}{t_{\max}}}-1}{e-1},$$

where *t_max_* is the maximum value of the number of iterations.

Bringing Equation (15) as in Equation (14), the following is obtained:

(16) $$M=\left(0.35+0.25\times \frac{e^{\frac{t}{t_{\max}}}-1}{e-1}\right)\times T\left(t\right),T\left(t\right)=e^{\frac{-t}{t_{\max}}},$$

The position update equation for the development path process is as follows:

(17) $$Z_{i}\left(t+1\right)=M\times G\left(t\right)+BM_{i}\left(t\right)⊗\left(\theta_{1}\times \left(G\left(t\right)-Z_{i}\left(t\right)\right)+\left(1-\theta_{1}\right)\times \left(\bar{Z}\left(t\right)-Z_{i}\left(t\right)\right)\right),$$

where, *θ*_1_ = 2 × *rand* − 1. *BM_i_*(*t*) is the Brownian random number vector. The sign ⊗ represents entry-wise multiplications. *G*(*t*) denotes the optimal solution. $\bar{Z}\left(t\right)$ is the location of the center of mass of the sample population with the following expression:

(18) $$\bar{Z}\left(t\right)=\frac{1}{N_{o}}\sum_{i=1}^{N_{o}}Z_{i}\left(t\right),$$

In summary, the complete position update equation for the SAO algorithm is as follows:

(19) $$\begin{array}{l} Z_{i}\left(t+1\right)=\left\{\begin{array}{l} Elite\left(t\right)+BM_{i}\left(t\right)⊗\left(\begin{array}{l} rand\times \left(G\left(t\right)-Z_{i}\left(t\right)\right)+\\ \left(1-rand\right)\times \left(\bar{Z}\left(t\right)-Z_{i}\left(t\right)\right) \end{array}\right),i\in z_{a}\\ \begin{array}{c} M\times G\left(t\right)+BM_{i}\left(t\right)⊗\left(\begin{array}{l} \theta_{1}\times \left(G\left(t\right)-Z_{i}\left(t\right)\right)+\\ \left(1-\theta_{1}\right)\times \left(\bar{Z}\left(t\right)-Z_{i}\left(t\right)\right) \end{array}\right),i\in z_{b} \end{array} \end{array}\right.\\ Elite(t)\in Best\left[G\left(t\right),Z_{2nd}\left(t\right),Z_{3rd}\left(t\right),Z_{50\%}\left(t\right)\right] \end{array},$$

**Figure 7:**
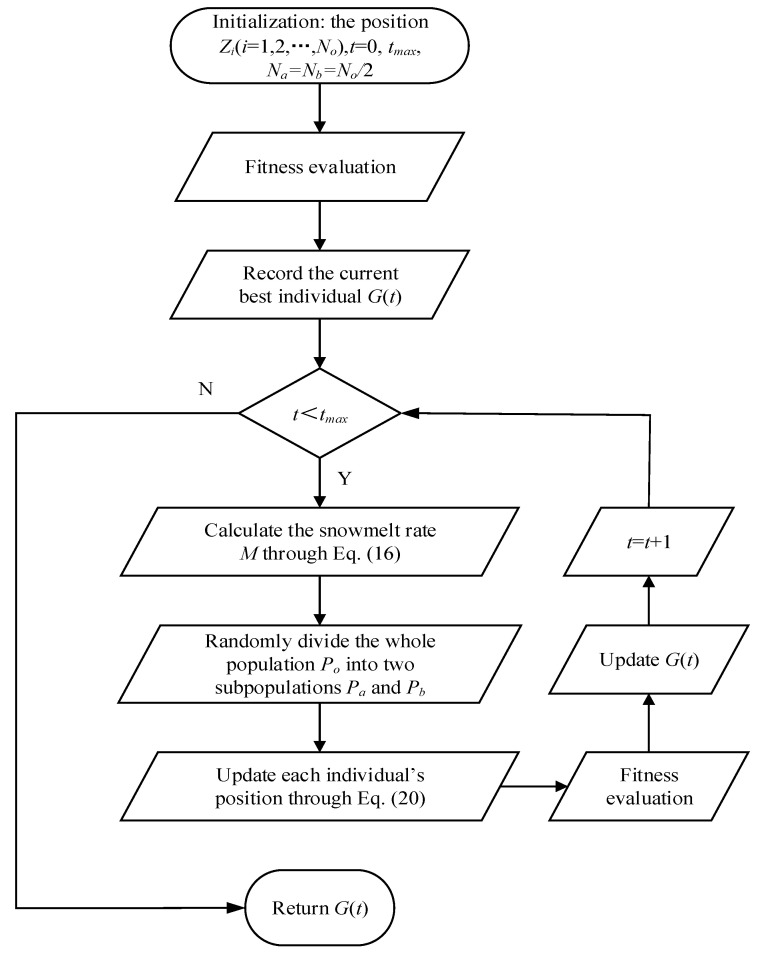
Iterative flowchart of the GVSAO algorithm.

After enhancing the SAO algorithm with the good point set algorithm and periodic oscillatory mutation policy, the complete position update equation for the GVSAO algorithm is as follows:

(20) $$\begin{array}{l} Z_{i}\left(t+1\right)=\left\{\begin{array}{l} \begin{array}{c} Z_{i}\left(t\right)\left[1+A\left(0.5-rand\right)\right],\delta =1\\ Elite\left(t\right)+BM_{i}\left(t\right)⊗\left(\begin{array}{l} rand\times \left(G\left(t\right)-Z_{i}\left(t\right)\right)+\\ \left(1-rand\right)\times \left(\bar{Z}\left(t\right)-Z_{i}\left(t\right)\right) \end{array}\right),\delta =0 \end{array},i\in z_{a}\\ \begin{array}{c} M\times G\left(t\right)+BM_{i}\left(t\right)⊗\left(\begin{array}{l} \theta_{1}\times \left(G\left(t\right)-Z_{i}\left(t\right)\right)+\\ \left(1-\theta_{1}\right)\times \left(\bar{Z}\left(t\right)-Z_{i}\left(t\right)\right) \end{array}\right),i\in z_{b} \end{array} \end{array}\right.\\ Elite(t)\in Best\left[G\left(t\right),Z_{2nd}\left(t\right),Z_{3rd}\left(t\right),Z_{50\%}\left(t\right)\right] \end{array},$$

where *Z*_2*nd*_(*t*) and *Z*_3*rd*_(*t*) represent the second-best individual and the third-best individual in the current population, respectively. *Z*_50%_(*t*) denotes the position of the center of mass of the individual whose fitness value is ranked in the top 50%. *z_a_* and *z_b_* denote the sizes of subpopulations *P_a_* and *P_b_*, respectively, in the iterative process. The iterative flowchart of the GVSAO algorithm is shown in Figure 7.

**Figure 8:**
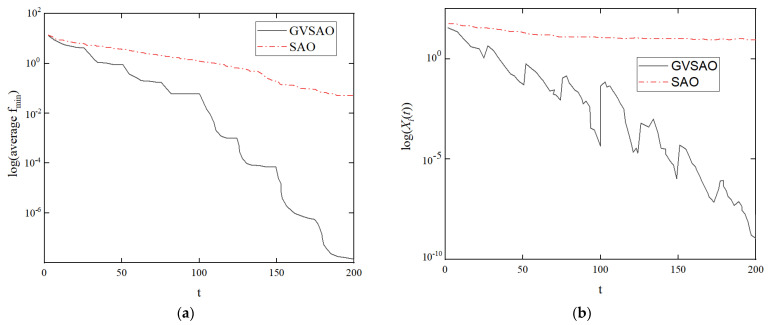
Comparative results of the different algorithms: (**a**) averaged best objective function values versus generations; (**b**) swarm diversity versus generations.

Comparison of the GVSAO in a simple case: The Ackley test function was selected as the benchmark test function. The Ackley test function is a multimodal test function. To reduce the computational cost, the problem dimension, d, was set to 3, the cluster size, s, to 5, and the maximum number of iterations, T, to a fixed value of 200 in the experiments. All algorithms were run 40 times, and the average results were calculated. The results are depicted in Figure 8. The averaged best objective function values versus generations are shown in Figure 8a. The GVSAO outperforms the SAO algorithm. It decreases the required generations by 50% or more compared with the SAO. Additionally, the accuracy of the solution is at about the 10^−7^ level. In Figure 8b, the swarm diversity versus generations is observed. The swarm diversity of the GVSAO gradually decreases during the generations. However, its change is in stages and periodic due to periodic mutation applications.

## 3. Test Step

**Figure 9:**
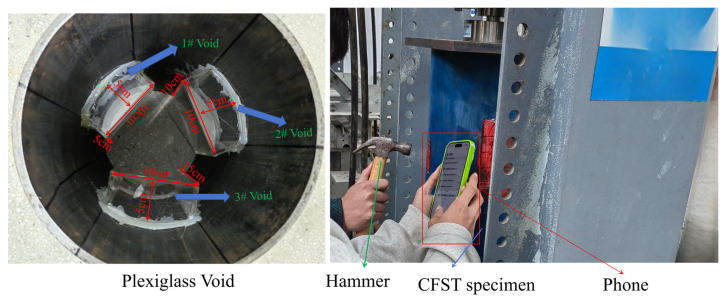
Experimental setup.

To verify the feasibility and effectiveness of the above algorithms, laboratory tests were conducted by digging voids of different sizes in the CFST specimens. As shown in Figure 9, the striking device was a hammer, the percussion signal recording device was a smartphone, and the source of the percussion sound was a CFST specimen. A hammer was used to apply an impact load of 30 ± 5 N. Data outside the range of 30 ± 5 N were excluded. The smartphone model was an iPhone 14, which features dual-frequency GPS positioning. Its frequency response range is 20 Hz ~ 20,000 Hz. Axial force was applied using a hydraulic servo loading system (Model MTS 810,manufactured by MTS Systems of the United States). Through closed-loop feedback via a pressure transducer (range: 0~2000 kN, accuracy: ±0.3%), the loading error was controlled to within ±1%. After testing, the load was unloaded to zero at a rate of 0.2 kN/s to prevent secondary damage to the component caused by impact loads.

**Figure 10:**
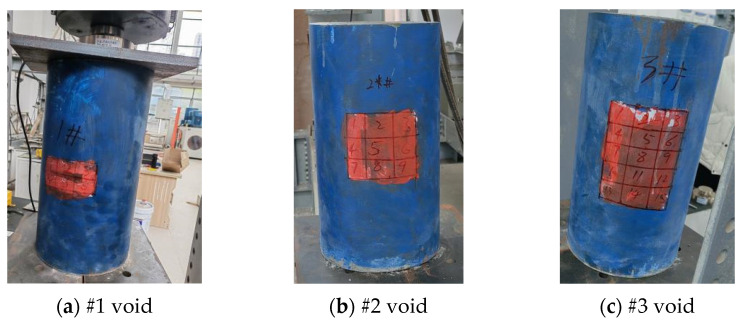
Void location marking.

The tube has a diameter of 220 mm, height of 400 mm, and thickness of 6 mm. The compressive strength of the filled concrete, *f_c_*, is 50 MPa. Three artificial voids made of plexiglass were placed near the inner surface of the steel tube before the concrete was poured, as shown in Figure 10. The size of #1 void is 100 mm × 50 mm × 50 mm (length × depth × height), the size of #2 void is 100 mm × 50 mm × 100 mm, the size of #3 void is 100 mm × 50 mm × 150 mm. The void is arranged with 3 mm plexiglass and glued to the inner wall of the steel tube with strong adhesive, and the position of the outer wall of the steel tube corresponding to the position of the void should be marked accordingly. The specimens were cured under standard conditions for 28 days before testing commenced. After the concrete is poured, mark the outside of the tubular at the location of the void, as shown in Figure 10.

The #1, #2, and #3 voids were divided into 6, 9, and 15 percussion areas, respectively. Additionally, five percussion areas were laid out at the no-void location, with each percussion area repeated for five impacts. As a result, a total of 225 signals were captured by the signal recording equipment and subsequently normalized to form the initial signal dataset. The enhanced signals are reconstructed by applying further normalization with the three data-enhancement techniques described in Section 2.1, which together with the normalized original signals constitute the reconstructed signal dataset. As shown in Table 2, this dataset was categorized using the four void heights of 0 mm, 50 mm, 100 mm, and 150 mm, which correspond to the respective sample labels.

**Figure 11:**
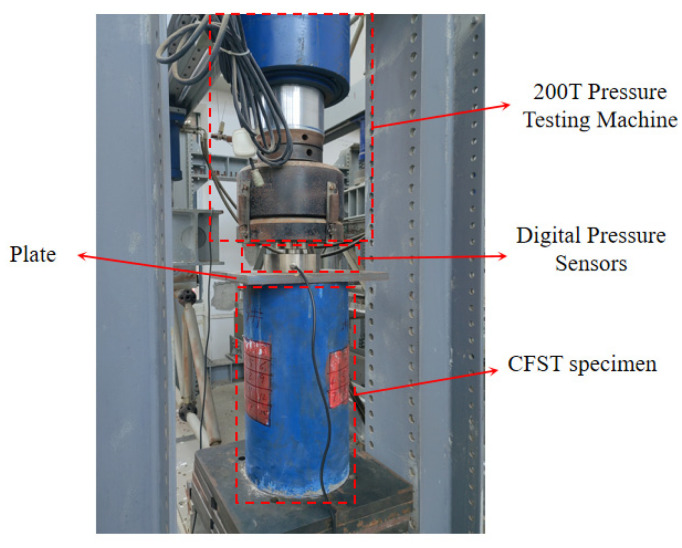
Pressure load test.

It is widely recognized that the main arch ring of the CFST bridge is dominated by axial pressure. The amplitude and frequency of the percussion sound vary under different pressure loads. During testing, the signal-to-noise ratio was maintained between −1 dB and 1 dB. To ensure consistency across recordings, the smartphone was positioned approximately 5 cm from the tapping source. To simulate this phenomenon, the following experimental procedure was designed:

(1) CFST specimens were tested by placing them in a pressure tester 28 days after the completion of the concrete placement, as shown in Figure 11. First, when the pressure was zero, each labeled void position was sequentially percussed with a hammer, and the resulting sound was recorded using a cell phone. For each percussion, a sample sound was recorded, and the corresponding sound file was appropriately labeled.
(2) Pressure was applied by the press, and when the pressure was 20 tons, the labeled position of each void was percussed sequentially with a hammer, and the sound was recorded with a cell phone. For each percussion, a sample sound was recorded, and the sound file was appropriately labeled.
(3) When the pressure was 50 tons, a hammer was used to percuss the labeled position of each void in turn, and a cell phone was used for sound recording. For each percussion, a sample sound was recorded, and the sound file was appropriately labeled.
(4) When the pressure was 100 tons, a hammer was used to percuss the labeled position of each void in turn, and a cell phone was used for sound recording. For each percussion, a sample sound was recorded, and the sound file was appropriately labeled.

## 4. Results and Discussion

### 4.1. CFST Void Detection Training Under Different Pressure Loads

In this section, the maximum pooling layer is applied to downscale the 2D time–frequency matrix to 1D time–frequency parameters. Table 3 shows the division of the original dataset and the enhanced signal dataset. Each dataset was first proportionally divided into a training set (80%) and a test set (20%). The training set is then further divided into a training set and a validation set for model fitting. In this section, the GVSAO algorithm is used to optimize the hyperparameters in the convolutional layer, which include the learning rate, convolutional kernel size, and the number of neurons. Table 4 shows the final values of the optimized hyperparameters.

To characterize the void percussion sound features of the CFST specimens under different pressure loads, the sound data are now grouped and numbered again, as shown in Table 5.

**Figure 12:**
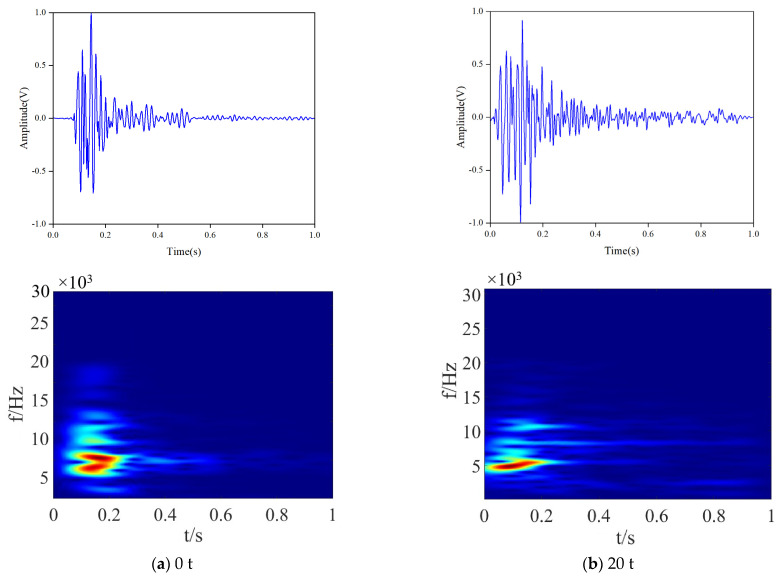

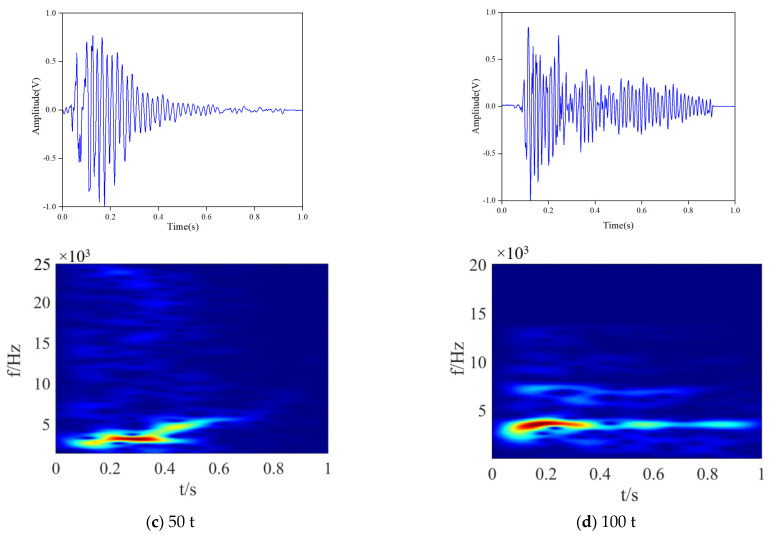
Diagrams of impact signals for defect sample #2 under different axial compression loads.

Related studies have been conducted to predict the audio of void percussion at different heights under unpressurized loading, and the prediction accuracy has reached more than 99% [33,34,35]. However, in practical engineering applications, the arch ring of the CFST arch bridge serves as the axial pressure member. Under the influence of different pressure loads, the sound emitted from the void area becomes notably “lower” in tone. To explain the differences in impact sounds under varying pressure loads, a time–frequency analysis was conducted. Taking the audio sample labeled #2 as an example, the time–frequency plots under different axial pressure loads are shown in Figure 12.

As shown in Figure 12, as the load increases, the main frequency of the acoustic wave first shifts toward the high-frequency range and then shifts toward the low-frequency range. This demonstrates that as stiffness increases, the natural frequency rises. When the axial load approaches the critical buckling value, the lateral stiffness of the specimen degrades, and the equivalent stiffness decreases, resulting in a decrease in the acoustic frequency.

### 4.2. Accuracy and Efficiency of Predictive Modeling

**Figure 13:**
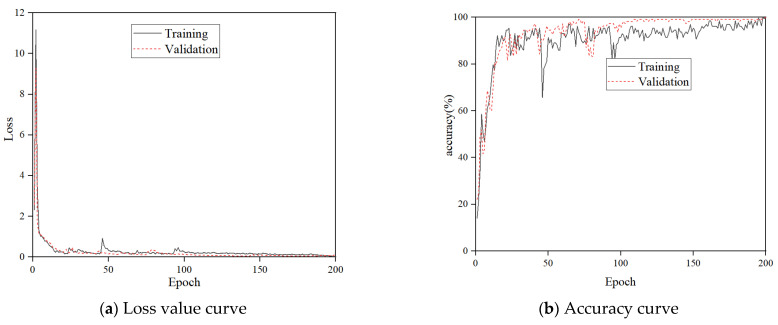
Plot of test results for the original dataset.

Due to the space limitation of the paper, the computational accuracy and efficiency of the prediction model demonstrated using sample data with a pressure load of 50 tons as an example. The model training was completed after 200 iterations. The accuracy and loss value curves for the original dataset test are shown in Figure 13. For better presentation, the accuracy results for each dataset under different pressure loads are listed in Table 6. Figure 13 shows the accuracy confusion matrix for the two tested datasets.

**Figure 14:**
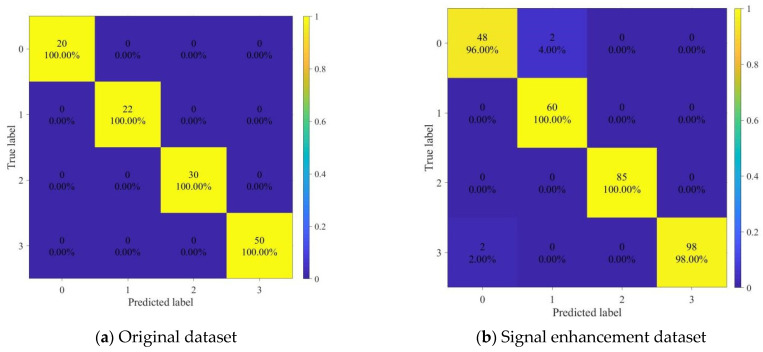
Confusion matrix of the accuracy of the test.

From Figure 13 and Figure 14 and Table 5, it can be seen that for the original dataset, the training set accuracy converges to about 99.89%, with a loss value of approximately 0.007. The prediction is 96.30%. The recall is 100%. The F1 score is 98.11%. The validation set accuracy converges to about 99.2%, with a loss value of approximately 0.04. The prediction is 95.60%. The recall is 97.44%. The F1 score is 95.25%. The final test accuracy was 100%. In contrast, for the signal enhancement dataset, the final test accuracy is 97.2%. The prediction is 94.51%. The recall is 99.36%. The F1 score is 96.56%.

### 4.3. Comparison of the Effectiveness of Different Algorithms in Optimizing the Training Model

To verify the advantages of the GVSAO algorithm proposed in this paper over other methods, four commonly used optimization algorithms were selected for comparative experimental analysis. These include the following: (1) XGBoost; (2) Random Forest (RF); (3) LightGBM. In this study, all comparison models (including XGBoost, Random Forest, and LightGBM) adopted a unified feature input standard. For audio data, MFCC features were first extracted, as these capture the key spectral characteristics of the audio signal. The two-dimensional MFCC features were then converted into one-dimensional feature vectors by averaging them over the time dimension, resulting in tabular data for input. The specific parameters for feature extraction were as follows: feature dimension M = 24, with 12 dimensions for the cepstral coefficients and 12 dimensions for the first-order differences. For high-frequency signals, a shorter frame length is required to capture transient features. Therefore, the length of each frame was set to 20 ms. The frame shift was set to half the frame length. Due to space limitations, the specific processing workflow will not be elaborated upon here. Table 7 shows the optimal hyperparameters for different algorithms.

**Figure 15:**
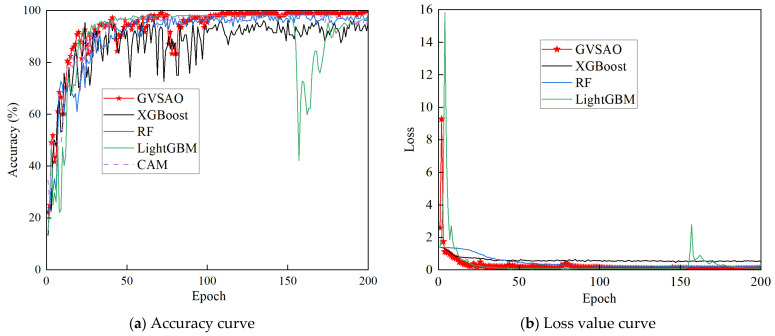
Comparison of results after training with different algorithms.

The accuracy-versus-loss value curves for the signal enhancement dataset under a 50-ton pressure load for the above algorithms’ training are plotted in Figure 15.

As can be seen from Figure 15, the proposed method performs superiorly in terms of classification accuracy in the training set, smoothing out to about 97.2% after convergence. After completing 200 iterations, the loss value minimized and converged to around 0.08. Both the convergence speed and the final convergence value outperformed other methods, indicating that the proposed method has the strongest data-fitting capability.

**Figure 16:**
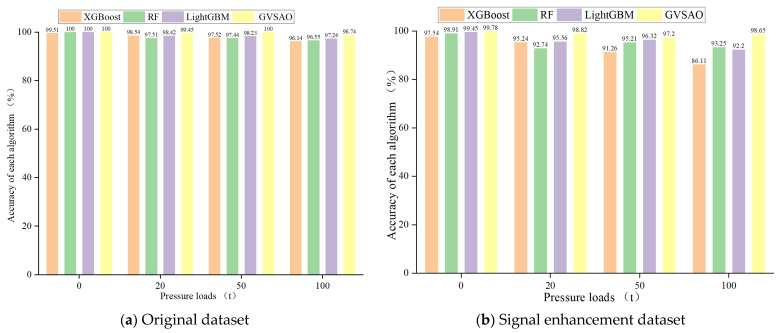
Testing the accuracy of each algorithm under different pressure loads.

As shown in Figure 16, the test accuracy of each algorithm for the two datasets under different pressure loads is presented.

As depicted in Figure 16, the original signal set was tested with high precision, with the accuracy ranging from a minimum of 96.14% to a maximum of 100%. This indicates that all algorithms exhibit excellent performance in processing signals without the addition of noise. In contrast, the test accuracy for the signal enhancement dataset ranges from a minimum of 86.11% to a maximum of 99.78%, which is relatively lower. Notably, the XGBoost algorithm has the lowest test accuracy of approximately 86.1%, because it fails to account for the temporal characteristics between data points at different time instances. The two methods RF and LightGBM are comparable, and both can preserve different temporal correlation feature information, thus enhancing the classification accuracy. However, RF can only capture the dependency of local temporal features and cannot achieve the sharing of temporal features on a global scale, which may lead to gradient vanishing in long sequences, resulting in a decrease in test accuracy to 92.7%. Although LightGBM solves the RF gradient vanishing problem to a certain extent and can process temporal features globally, it suffers from high computational complexity, slow computation speed, and demanding hardware requirements, making it difficult to achieve optimal results for long sequences, leading to a reduction in test accuracy to 92.2%. As a result, the average test accuracy of RF and LightGBM is only approximately 95%. The performance of the validation set was unstable during the training process.

This study conducted a total of 200 full training epochs during the model training stage. A batch size of 64 was used during training. The compute nodes used for model training and evaluation were equipped with two NVIDIA Tesla V100 GPUs (each with 16 GB of HBM2 memory). The CPU was a 14th generation Intel^®^ Core™ i5–14600 KF (2.5 GHz, 14 cores, 20 threads), with 128 GB of DDR4 system memory and 1 TB of NVMe SSD storage.

### 4.4. Robustness Analysis

In the face of noise interference at the construction site, it is necessary to maintain high diagnostic accuracy to illustrate the effectiveness of the method proposed in this paper. Therefore, the test set samples are fed into each trained model by adding construction site noise interference to verify the robustness of the proposed methods, respectively.

To further evaluate the ability of the proposed method to enhance SNR, Gaussian white noise with SNRs of −10 dB, −5 dB, 0 dB, 5 dB, and 10 dB (the lower the SNR, the higher the relative noise) was selected as the input samples of the test set, which were then processed by the optimal models saved during training using various methods. The prediction accuracy when the pressure load is 50 tons is taken as an example for robustness analysis, and the accuracy of the model prediction of each algorithm under different SNRs is obtained, as shown in Table 8.

As can be observed from Table 8, the test accuracy of the method proposed in this paper declines by 2.15% and 3.22% under Gaussian white noise conditions of −10 dB and −5 dB, respectively. Specifically, the test accuracies of the XGBoost and RF methods experience a significant decrease, while the LightGBM method shows relatively smaller reductions in comparison. Nevertheless, the overall testing performance of these methods still lags behind that of the method proposed in this paper. This indicates that the optimization algorithm introduced in this paper sustains a high level of diagnostic classification accuracy even when faced with varying degrees of construction site noise interference, thus demonstrating its strong noise suppression ability and robust diagnostic performance.

## 5. Discussion

This study proposes a CFST-based void detection method that combines the GVSAO algorithm with a dual-channel parallel convolutional neural network (CNN). Regarding acoustic signal feature processing, this study focuses on using the GVSAO algorithm to simultaneously optimize network hyperparameters (learning rate and regularization coefficient) and feature extraction parameters (GAF window size and sampling frequency). It effectively avoids the problem of getting stuck in local optima, which is common with traditional methods, significantly improving the efficiency of global optimization for parameter combinations. By introducing a nonlinear decay factor and a fitness feedback mechanism, the study addresses the issue of PSO getting trapped in local optima during the later stages of the iterative process. In terms of experiments, this study conducted cavity detection under different axial pressures from the perspective of the stress distribution in CFST arch bridges. It provides more reliable technical support for the non-destructive testing of internal defects in CFST structures in practical engineering applications. Using acrylic sheets affixed to the inner steel walls to simulate voids still does not accurately replicate the reflection of sound waves in reality.

The dataset used in this study is a small-scale laboratory dataset. The sample size is limited, and the data distribution may differ from that of actual complex engineering environments. This limits the generalizability and extrapolation potential of the research results to some extent. Second, the artificially created voids used in the experiments differ significantly from naturally occurring defects in actual engineering environments in terms of morphological characteristics, distribution patterns, and interactions with the surrounding medium. Artificial voids typically have regular shapes and well-defined boundaries, whereas real-world defects may be more complex and irregular. This may result in the reduced performance of models trained and validated using artificial void data when applied in practical scenarios.

## 6. Conclusions

To enhance the accuracy of void percussion detection in CFST arch bridges under different SNRs, this study proposes a CFST void diagnosis method that integrates a GVSAO with a parallel CNN. An accurate voiding diagnostic model was successfully developed. The main findings of this study are summarized as follows:

(1) GAF is utilized to image the percussive sound signal to construct a dual-channel parallel CNN structure. The GAF is decomposed into a GASF map and GADF map, which are simultaneously fed into the CNN for training. Subsequently, the outputs of the two channels are spliced and fused. Finally, the GVSAO algorithm is utilized for classification. This method ensures that both the temporal and spatial features of the percussion sound signal are fully preserved, while the interference of different construction noises is effectively avoided.
(2) To enhance the robustness of the diagnostic model to external disturbances, this study records and integrates three types of on-site construction noises—mechanical equipment noise, wind noise, and welding noise—and reconstructs them into a signal-enhanced dataset. Given that the CFSTs in arch bridges is under full-section pressure for long periods, the percussion sound samples of the CFST specimens under pressure loads of 0 ton, 20 tons, 50 tons, and 100 tons are recorded. Finally, by labeling, the original dataset and signal enhancement dataset of the void percussion signals under different pressure loads are composed.
(3) Using the GVSAO algorithm with the dual-channel parallel CNN prediction model, the test accuracies on the original signal dataset exceeded 98.74%. Notably, the accuracy reached 100% for pressure loads of 0 tons and 50 tons. In addition, the test accuracies on the signal enhancement dataset exceeded 97.2%. This shows that the model still maintains a high level of classification, indicating that it can suppress noise significantly and has excellent robustness.
(4) The model that integrates the GVSAO algorithm with a parallel CNN has the advantage of higher prediction accuracy compared with other traditional machine learning and deep learning algorithms after iterative training parameter optimization. It outperforms algorithms such as XGBoost, RF, and LightGBM in terms of diagnostic robustness. Furthermore, in the presence of noise interference, the proposed model maintains a diagnostic accuracy of over 96%, thereby validating its effectiveness and strong anti-interference capability.

## Figures and Tables

**Table 1:** Parameters of the dual-channel parallel CNN network.

Layer (Type)	Output Shape	Connected To
Input_1 (InputLayer)	(None, 227, 227, 3)	
Input_2 (InputLayer)	(None, 227, 227, 3)	
conv2d (Conv2D)	(None, 115, 115, 64)	input_1
conv2d_1 (Conv2D)	(None, 115, 115, 64)	input_2
max_pooling2d	(None, 58, 58, 64)	conv2d
max_pooling2d_1	(None, 58, 58, 64)	conv2d_1
flatten_1 (Flatten)	(None, 120)	max_pooling2d
flatten_2 (Flatten)	(None, 120)	max_pooling2d_1
Concatenate	(None, 240)	flatten, flatten_1

**Table 2:** Datasets with different heights of voids.

Void Height (mm)	Void Scope (mm)	Label Number	Percussion Number	Data Augmentation
0	0 × 0	0	25	75
50	100 × 50	1	30	90
100	100 × 50	2	45	135
150	100 × 50	3	75	225

**Table 3:** Dataset partitioning of the raw and enhanced signal dataset.

Dataset Index	Training Set	Validation Set	Testing Set	Total Number
Raw dataset	540	180	180	900
Enhanced signal dataset	2160	720	720	3600

**Table 4:** Values of hyperparameters.

Hyperparameters	Learning Rate	Convolutional Kernel Size	Number of Neurons
Upper limit	0.01	5	120 900
Lower limit	0.001	1	1
Final values	0.003570254	3	120

**Table 5:** Datasets for the void detection under different pressure loads.

Pressure Loads (t)	Raw Dataset	Enhanced Signal Dataset
0	900	3600
20	900	3600
50	900	3600
100	900	3600

**Table 6:** Dataset partitioning of the raw and enhanced signal dataset.

Dataset Index	0 t	20 t	50 t
Raw dataset	100	99.45	100
Enhanced signal dataset	99.78	98.82	97.20

**Table 7:** The optimal hyperparameters for different algorithms.

Model	Hyperparameter
XGBoost	learning rate = 0.1, n_estimators = 100, max depth = 3 = 5, min_num_neurons = 150
RF	max depth = 3, n_estimators = 150, min_samples_leaf = 7 min_samples_leaf = 7
LightGBM	learning rate = 0.1, n_estimators = 50, num_leaves = 8 num_leaves = 8
GVSAO	learning rate = 0.01, size_kernel = 5, max_iter = 10, min_num_neurons = 120, num_folds = 10

**Table 8:** Prediction accuracy of the optimized models for each algorithm with different SNRs (%).

Algorithm Type	SNR				
	−10 dB	−5 dB	0 dB	5 dB	10 dB
XGBoost	89.54	92.56	97.52	98.45	98.48
RF	93.78	94.23	97.44	98.65	98.87
LightGBM	93.51	95.55	98.23	98.98	99.54
GVSAO	96.78	97.85	100	100	100

## Data Availability

The original contributions presented in this study are included in the article. Further inquiries can be directed to the corresponding author.
